# Case Report: Synchronous lingual osseous choristoma and suprahyoid thyroglossal duct cyst: insights into the embryological thyroglossal-hyoid axis

**DOI:** 10.3389/fsurg.2026.1856644

**Published:** 2026-07-17

**Authors:** Hyun Je Kim, Sun-Young Jun, Ho-Kyung Lim

**Affiliations:** 1 Department of Oral and Maxillofacial Surgery, Incheon St. Mary’s Hospital, Incheon, Republic of Korea; 2 Department of Pathology, Incheon St. Mary’s Hospital, College of Medicine, The Catholic University of Korea, Incheon, Republic of Korea; 3 Department of Oral and Maxillofacial Surgery, Korea University Guro Hospital, Seoul, Republic of Korea

**Keywords:** case report, embryology, endoscopic surgery, hyoid bone, lingual osseous choristoma, thyroglossal duct cyst, tongue base

## Abstract

Lingual osseous choristoma is an exceedingly rare benign lesion characterized by the presence of mature bone tissue within the soft tissue of the tongue, where osseous structures are not normally found. Thyroglossal duct cysts (TGDCs) are common congenital midline neck lesions resulting from incomplete involution of the thyroglossal duct during embryogenesis. Both entities share a common embryological pathway extending from the tongue base through the hyoid bone to the lower cervical region. However, the synchronous occurrence of a lingual osseous choristoma and a suprahyoid TGDC in a single patient has rarely been reported. A 26-year-old woman presented with a slowly enlarging posterior tongue mass and a persistent foreign-body sensation during swallowing. Intraoral examination revealed a firm, pedunculated lesion covered by intact mucosa at the midline posterior tongue near the circumvallate papillae. Contrast-enhanced cervical magnetic resonance imaging incidentally demonstrated a well-circumscribed cystic lesion posterior to the hyoid bone, suggestive of a suprahyoid TGDC. Under general anesthesia, the lingual mass was excised via an endoscope-assisted intraoral approach, followed by transcervical excision of the TGDC with partial segmental resection of the hyoid bone and *en bloc* removal of the thyroglossal duct tract extending approximately 5 cm superiorly. Histopathological examination confirmed the diagnosis of lingual osseous choristoma, showing mature lamellar bone covered by stratified squamous epithelium, and a thyroglossal duct cyst with characteristic cyst wall features. The patient recovered without complications, with intact swallowing function and no recurrence at 18 months. This case suggests that lingual osseous choristoma and suprahyoid TGDC may represent dual manifestations of residual mesenchymal tissue along the thyroglossal duct-hyoid embryological axis, giving rise to osseous and cystic lesions at distinct anatomical levels. These findings raise the possibility of an embryological link between posterior lingual osseous lesions and the thyroglossal duct system, highlighting the need for thorough imaging of the entire thyroglossal tract when encountering osseous lesions of the tongue base.

## 1 Introduction

Thyroglossal duct cyst (TGDC) arises from incomplete involution of the thyroglossal duct, which originates at the foramen cecum on the dorsal surface of the tongue and descends along the midline of the neck to the anterior aspect of the thyroid gland during embryologic development. Although the thyroglossal duct normally regresses by approximately the 10th week of gestation, persistent epithelial remnants may remain anywhere along this migratory pathway, forming cysts or fibrous tracts extending from the tongue base to the thyroid region (1–4). TGDC is one of the most common congenital midline neck masses, typically diagnosed in children and adolescents, with the highest prevalence around the hyoid bone. When TGDC occurs in atypical locations such as within the tongue or confined to the tongue base, it may present with nonspecific oral or pharyngeal symptoms, leading to delayed diagnosis (1, 3, 5).

Lingual osseous choristoma is an exceptionally rare benign lesion characterized by the presence of mature bone tissue within the soft tissues of the tongue. It most commonly arises in the posterior one-third of the tongue, particularly in the midline region adjacent to the foramen cecum and circumvallate papillae, and typically presents as a firm, pedunculated, mucosa-covered mass in young women (6–8). The etiopathogenesis of lingual osseous choristoma is not fully understood; two main theories have been put forward: an embryological hypothesis involving persistence of osteogenic primordia from the branchial arch and hyoid apparatus, and a reactive hypothesis involving BMP-mediated osseous metaplasia (6, 9).

The anatomical course of the thyroglossal duct closely overlaps with the tongue base region where osseous choristoma most frequently arises. This anatomical overlap led us to consider whether both lesions might share a common embryological origin. As far as we are aware, no prior report has described the synchronous occurrence of lingual osseous choristoma and TGDC in the same patient. We present such a case and discuss the potential shared embryological basis, surgical management, and clinical implications.

## 2 Case description

The clinical course is summarized in Table 1.

**Table 1:** Timeline of the clinical course.

Time Point	Patient Age	Clinical Event
Adolescence	Approximately 10–18 years	Patient first noticed a small, asymptomatic nodule at the posterior tongue base
4 years before presentation	Approximately 22 years	Gradual enlargement of the tongue-base mass began, accompanied by increasing foreign-body sensation and dysphagia during solid food intake
Initial presentation	26 years	Patient referred for evaluation of a slowly enlarging posterior tongue mass
Preoperative imaging	26 years	MRI and CT revealed a posterior tongue mass and an additional cystic lesion posterior to the hyoid bone, suggestive of suprahyoid TGDC
Surgery	26 years	Endoscope-assisted intraoral excision of the lingual mass and transcervical TGDC excision with partial hyoid bone resection were performed
Postoperative period	26 years	A posterior pharyngeal wall defect (caused by dense cyst adhesion) was repaired with double-layer closure; a temporary L-tube was placed for airway protection and repair-site stabilization
Follow-up	18 months after surgery	No recurrence or functional impairment observed; continued long-term surveillance planned

The table presents the chronological clinical course of the patient, including the approximate age at initial recognition of the posterior tongue-base nodule, symptom progression, preoperative imaging findings, surgical treatment, postoperative management, and 18-month follow-up. The term “adolescence” refers to approximately 10–18 years of age, and the onset of gradual enlargement occurred approximately 4 years before presentation, when the patient was around 22 years old. The posterior pharyngeal wall defect encountered during surgery was considered to be related to dense adhesion of the retro-hyoid cystic tract and was managed with double-layer closure and temporary L-tube placement. No recurrence or functional impairment was observed at 18 months after surgery, although continued long-term surveillance was planned.

### 2.1 Clinical findings

A 26-year-old woman was referred to the Department of Oral and Maxillofacial Surgery with a chief complaint of a slowly enlarging mass at the posterior aspect of the tongue accompanied by a foreign-body sensation during swallowing. The patient reported that she had been aware of a small nodule at the tongue base since adolescence (approximately 10–18 years of age); however, the lesion had been asymptomatic and stable in size until approximately 4 years prior to presentation (age 22), when gradual enlargement was noted. From that time, she experienced increasing discomfort and dysphagia, particularly during solid food intake. She denied dyspnea, voice changes, weight loss, or systemic symptoms.

She had no significant past medical history and no prior surgery, trauma, or radiotherapy to the head and neck. There was no relevant family history. She was a non-smoker and consumed alcohol occasionally.

Intraoral examination revealed a well-circumscribed, firm, pedunculated mass located at the midline of the posterior tongue base, adjacent to the circumvallate papillae. The lesion was covered by intact mucosa with normal coloration and showed no ulceration or bleeding. Palpation elicited no tenderness, and the mass was relatively mobile and distinct from the surrounding tongue musculature. No palpable cervical masses or lymphadenopathy were detected.

### 2.2 Radiologic evaluation

Contrast-enhanced magnetic resonance imaging (MRI) of the neck demonstrated a small pedunculated nodular lesion at the midline posterior tongue base without evidence of muscular invasion. An additional well-defined, unilocular cystic lesion was identified in the posterior midline region of the hyoid bone, closely abutting its posterior surface and located along the typical suprahyoid tract of the thyroglossal duct. The lesion exhibited high signal intensity on T2-weighted images with a thin enhancing wall, findings consistent with a TGDC (Figures 1A–F). Subsequent contrast-enhanced neck CT and facial bone CT confirmed both lesions. No evidence of airway compromise, thyroid abnormalities, or cervical lymphadenopathy was observed (Figures 1G–I).

**Figure 1:**
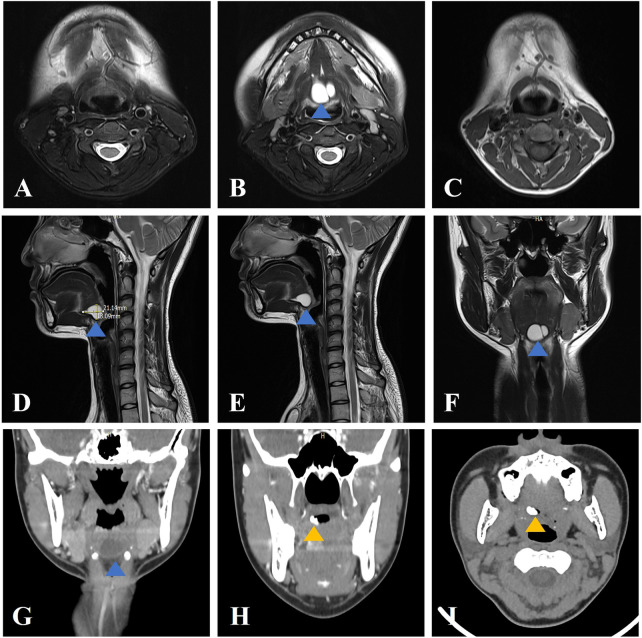
Preoperative imaging findings of the suprahyoid thyroglossal duct cyst and lingual osseous choristoma (blue arrow: thyroglossal duct cyst, yellow arrow: lingual osseous choristoma). **(A–C)** Preoperative contrast-enhanced neck MRI (axial views) demonstrating a well-circumscribed, cystic lesion (maximal diameter approximately 2 cm) in the midline suprahyoid/retrohyoid region. **(D,E)** Preoperative contrast-enhanced neck MRI (sagittal views) and **(F)** coronal view depicting the cystic lesion's location and its close anatomical relationship to the posterior aspect of the hyoid bone. **(G,H)** Preoperative contrast-enhanced neck CT (coronal reconstructions) and **(I)** axial reconstruction showing a small, well-defined hyperdense calcified lesion at the midline posterior tongue base near the foramen cecum/circumvallate papillae, consistent with lingual osseous choristoma, alongside the concurrent suprahyoid cystic tract.

### 2.3 Surgical management

Given the patient's worsening symptoms and the known risks of untreated TGDC—including infection and potential airway compromise—we planned surgical excision under general anesthesia. We used a combined approach, performing endoscope-assisted intraoral excision of the tongue base mass and transcervical excision of the TGDC with partial hyoid bone resection (Figures 2A–C).

**Figure 2:**
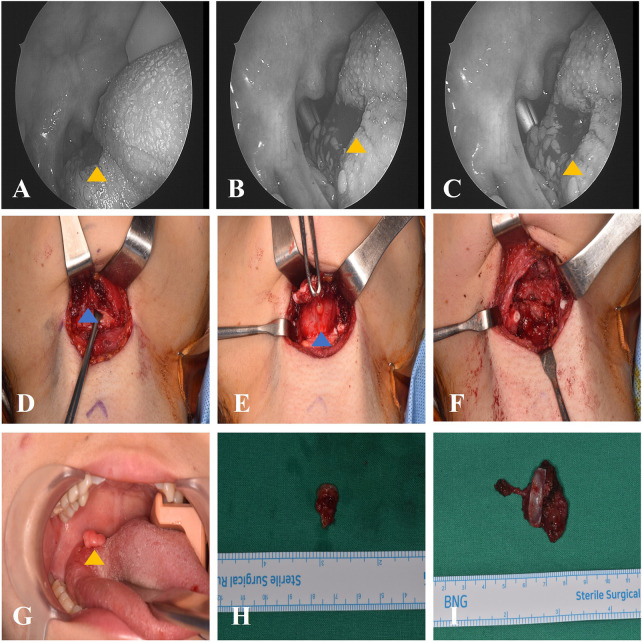
Intraoperative findings and gross specimens. (Blue arrow: Thyroglossal duct cyst, Yellow arrow: The excision site of the lingual osseous choristoma). **(A)** Endoscopic view of the posterior tongue base showing a well-defined, firm, pedunculated mass located at the midline near the circumvallate papillae. **(B)** Endoscopic exposure of the stalk during dissection using electrocautery. **(C)** Endoscopic view of the operative field immediately after complete intraoral excision of the lingual mass, demonstrating primary mucosal closure. **(D)** Transcervical exposure of the hyoid bone via a midline cervical incision, showing the cystic lesion tightly adherent to its posterior surface. **(E)** Mobilization and isolation of the retrohyoid thyroglossal duct cyst tract extending superiorly. **(F)** Surgical field following a modified Sistrunk procedure with partial segmental resection of the hyoid body and *en bloc* removal of the tract. **(G)** Clinical intraoral photograph demonstrating the preoperative position of the pedunculated tongue base mass. **(H)** Gross specimen of the excised lingual osseous choristoma, measuring approximately 0.8 cm. **(I)** Gross specimen of the excised thyroglossal duct cyst attached *en bloc* to the excised segment of the hyoid bone and the extended 5 cm tract.

After nasotracheal intubation, endoscopic visualization of the tongue base revealed a well-defined pedunculated mass at the midline posterior tongue base, just posterior to the circumvallate papillae, without local infiltration. The stalk was excised with an adequate safety margin using electrocautery and sharp dissection, and the mucosa was closed primarily with absorbable sutures (Figures 2G–I).

Subsequently, a midline cervical incision exposed the hyoid bone. A cystic lesion was identified on the posterior aspect of the hyoid, tightly adherent to the bone. Partial segmental resection of the hyoid bone was performed, and the thyroglossal duct tract was traced superiorly toward the tongue base and excised *en bloc* to a length of approximately 5 cm—a modified Sistrunk operation with extended suprahyoid tract removal (Figures 2D–F).

During dissection, a small defect occurred in the posterior pharyngeal wall adjacent to the hyoid region. This was directly attributable to the dense fibrous adhesion of the TGDC to the posterior pharyngeal wall, which prevented a clean tissue plane and necessitated sharp dissection in a confined space. The defect was immediately recognized and repaired with a double-layer closure involving both mucosal and muscular layers. To protect the airway and provide mechanical stabilization of the repair site, a temporary laryngeal tube (L-tube) was placed. The surgical site was closed in layers with placement of a drain.

### 2.4 Postoperative course

The patient recovered without complications. There was no airway compromise or notable hemorrhage. A video fluoroscopic swallowing study (VFSS) was performed to assess swallowing function and integrity of the pharyngeal repair; no aspiration, dysphagia, or residue was identified (Figures 3A–F). The L-tube and surgical drain were removed sequentially after confirmation of airway stability and satisfactory wound healing. The patient was subsequently discharged in stable condition. At 18-month follow-up, no evidence of recurrence, functional impairment, or residual symptoms was observed in either the tongue base or cervical region (Figures 3G–I).

**Figure 3:**
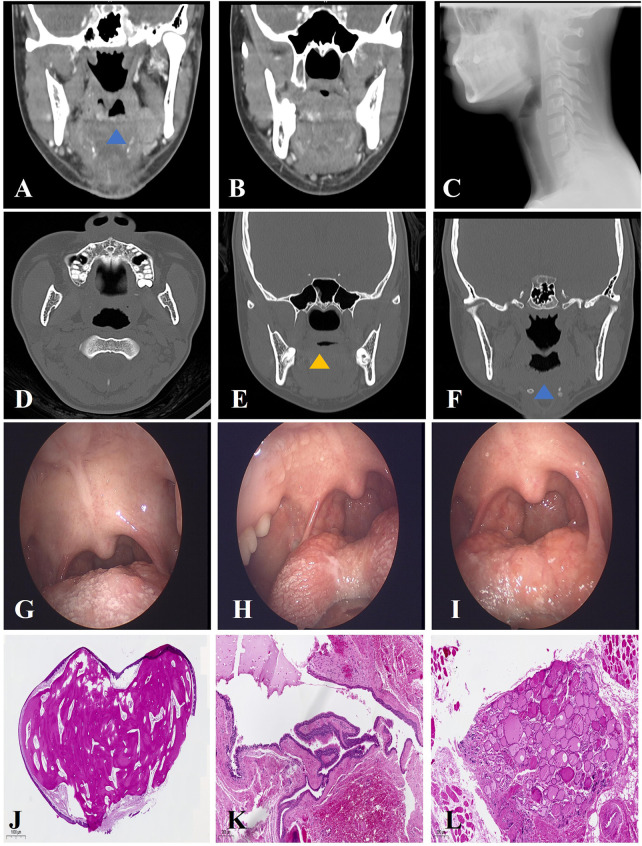
Postoperative follow-up at 18 months and gross and microscopic findings (blue arrow: absence of recurrence at the surgical site of the thyroglossal duct cyst, yellow arrow: No recurrence at the excision site of the lingual osseous choristoma). **(A,B)** Postoperative contrast-enhanced neck CT (soft-tissue window, coronal views) at 18-month follow-up showing no evidence of recurrent cystic lesions in the retrohyoid or suprahyoid tract. **(C)** Postoperative lateral neck radiograph (x-ray) demonstrating intact airway patency and the postoperative state of the hyoid bone. **(D)** Postoperative axial CT scan (bone window) and **(E,F)** coronal CT scans (bone window) confirming complete removal of the tongue base calcified mass and demonstrating the stable defect gap after partial segmental resection of the hyoid body without long-term structural complications. **(G–I)** Follow-up endoscopic examinations of the pharynx and tongue base at 18 months, demonstrating a completely healed, intact mucosa around the foramen cecum, circumvallate papillae, and lingual tonsils with no visual evidence of tumor recurrence. **(J)** Histopathological examination of the tongue nodule showing mature lamellar bone with well-developed Haversian systems beneath a normal stratified squamous mucosa, establishing the diagnosis of lingual osseous choristoma (H&E, original magnification ×1.8). **(K)** Histopathological section of the retrohyoid cyst showing a unilocular cyst wall lined by pseudostratified ciliated columnar epithelium with a fibrous connective tissue wall (H&E, ×7.2). **(L)** High-power view of the adjacent stroma within the cyst wall demonstrating ectopic normal thyroid follicles, confirming the definitive diagnosis of a thyroglossal duct cyst (H&E, ×6.8).

### 2.5 Histopathological findings

Gross examination of the excised tongue lesion revealed a firm, ovoid nodule measuring 0.8 cm in greatest dimension. Histologically, the lesion showed mature lamellar bone with well-developed Haversian systems beneath squamous mucosa, consistent with lingual osseous choristoma (Figure 3J). The excised posterior hyoid cyst was unilocular and lined by pseudostratified ciliated epithelium with a fibrous connective tissue wall (Figure 3K). Thyroid follicles were identified in the adjacent stroma (Figure 3L), confirming the diagnosis of TGDC.

## 3 Discussion

### 3.1 Key findings of this case

We report a rare concurrence of lingual osseous choristoma and retrohyoid TGDC in the same patient. This coexistence raises the possibility that both lesions arise from a shared embryologic source—residual mesenchymal tissue of the thyroglossal duct–branchial arch complex (2, 3, 10). Lingual osseous choristoma most commonly arises in the posterior one-third of the tongue, particularly in the midline region adjacent to the foramen cecum and circumvallate papillae. Histologically, it is characterized by mature lamellar bone covered by normal stratified squamous epithelium. Most reported cases share consistent clinical features, including female predominance, occurrence in young individuals, and presentation as a small, firm, pedunculated mass (9, 11, 12), all of which were consistent with our patient.

### 3.2 Comparison with previously reported cases

To place this case in context, we searched PubMed, Google Scholar, and SciSpace using the terms “lingual osseous choristoma,” “thyroglossal duct cyst,” “synchronous,” “concurrent,” and “tongue base.” No previously documented case of this specific synchronous combination was identified; to our knowledge, this is the first such report.

In related coexistence reports, Madana et al. described a 7-year-old girl with simultaneous TGDC and lingual thyroid in the absence of an orthotopic thyroid gland, treated successfully with a Sistrunk procedure with no recurrence at 18-month follow-up (13). A neuroglial choristoma within a TGDC has also been reported in a 2-year-old girl, demonstrating that choristomatous tissue can coexist with TGDC pathology, albeit of a non-osseous subtype. Thompson et al., in a large clinicopathologic series of 685 thyroglossal duct remnant cysts, noted extensive anatomical variability in TGDC location and histology, but no synchronous osseous choristoma was identified (14). The key distinguishing features of the present case are: (1) the osseous (rather than thyroid or neuroglial) nature of the choristoma; (2) the retrohyoid location of the TGDC; and (3) successful combined surgical management in a single operative session with preservation of swallowing function.

### 3.3 Evidence for and against the embryologic hypothesis

The etiopathogenesis of lingual osseous choristoma is explained by two principal hypotheses. The embryological hypothesis proposes that osteogenic or chondrogenic primordia derived from the second to fourth branchial arches and the hyoid apparatus persist near the tongue base and subsequently ossify. The posterior tongue, foramen cecum, and hyoid bone anatomically overlap with the thyroglossal duct migration pathway, creating a plausible environment for ectopic tissue persistence.

The reactive or metaplastic hypothesis suggests that chronic mechanical stimulation or microinflammation induces osteogenic differentiation of pluripotent mesenchymal cells via bone morphogenetic protein (BMP-2 and BMP-4) signaling (9, 15–18).

In our patient, both hypotheses may apply: mesenchymal cells near the lingual thyroglossal duct segment, subjected to repetitive mechanical stress, may have differentiated into mature bone, while epithelial remnants at the retrohyoid level formed a cyst. Thyroglossal duct remnants are present in up to 7% of the population, with 0.6%–3% located in the tongue or tongue base (12, 19).

This remains speculative, as we did not obtain immunohistochemical or molecular data. Studies using osteogenic markers such as RUNX2 and osteocalcin, or comparative genomic analyses, would be needed to test this hypothesis directly.

### 3.4 Differential diagnosis of tongue base and midline neck masses

Accurate preoperative diagnosis is important because tongue base masses encompass a wide range of benign and malignant entities with overlapping clinical features (20, 21). The most clinically relevant differential diagnoses include:

1. Lingual thyroid: The most critical entity to exclude; inadvertent excision may cause permanent hypothyroidism. Characterized by absence of orthotopic thyroid tissue on ultrasound or radionuclide scan (1, 20).
2. Dermoid/epidermoid cyst: Well-circumscribed cystic lesion with fat or keratinous debris on CT; high T1 signal on MRI (21).
3. Vallecular cyst: Smooth, thin-walled unilocular cyst adjacent to the epiglottis; presents with dysphagia and muffled voice (21).
4. Plunging ranula: Pseudocyst from the sublingual gland with characteristic “tail sign” on MRI (21).
5. Lymphatic malformation: Multiloculated cystic lesion with thin septations and no internal vascularity on Doppler; often presents in early childhood (21).
6. Lingual tonsil hypertrophy: Bilateral symmetric soft-tissue thickening without a discrete nodular lesion (20).
7. Minor salivary gland tumor/ossifying fibromyxoid tumor: Solid or calcified masses requiring histopathological confirmation (7, 20).

In the present case, CT evidence of a calcified lesion at the midline tongue base combined with histopathological confirmation of mature lamellar bone established lingual osseous choristoma. The concurrent suprahyoid cystic lesion was distinguished by its T2 hyperintensity and thin enhancing wall, consistent with TGDC (1, 10).

### 3.5 Surgical considerations: hyoid bone management and intraoperative complication

The posterior lingual osseous lesion was safely excised using an endoscope-assisted intraoral approach, which provided adequate visualization in an anatomically constrained region. Endoscopic approaches for tongue-base TGDCs have gained traction as effective minimally invasive alternatives (22). For the retrohyoid TGDC, a modified Sistrunk procedure was performed, including partial resection of the hyoid bone and meticulous superior tract excision extending beyond 5 cm. Complete removal of the tract is essential to minimize the risk of recurrence (17, 18).

A critical intraoperative decision involved the management of the hyoid bone after partial segmental resection. Two principal approaches have been reported: (1) simple suspension, in which the cut ends of the hyoid bone are left to heal naturally without formal reconstruction; and (2) formal reconstruction, using titanium plates, mesh, or absorbable fixation devices to restore hyoid continuity (23, 24). Proponents of reconstruction argue that restoring hyoid continuity preserves the biomechanical function of the hyoid as a suspension point for the tongue musculature and laryngeal framework, reducing the risk of postoperative dysphagia and laryngeal descent (23). However, the majority of published series report that simple suspension after limited hyoid resection during the Sistrunk procedure is well tolerated, with no significant long-term functional sequelae, provided that the greater horns are preserved (17, 18, 24). In the present case, partial segmental resection of the hyoid body was performed with preservation of the greater horns and bilateral suprahyoid muscle attachments. Given the confirmed integrity of swallowing function on VFSS and absence of postoperative dysphagia, we did not feel formal hyoid reconstruction was necessary. However, in cases requiring more extensive hyoid resection—particularly when both greater horns must be sacrificed—reconstruction should be considered to preserve laryngeal support and swallowing function (23).

A small defect in the posterior pharyngeal wall occurred during dissection, attributable to the dense fibrous adhesion between the cyst wall and the posterior pharyngeal mucosa. This risk should be communicated to patients during preoperative counseling, particularly in retrohyoid TGDC cases. The defect was immediately repaired with a double-layer closure of mucosal and muscular layers. A temporary L-tube was placed for airway protection and mechanical stabilization of the closure while edema resolved, and was removed after VFSS confirmed the integrity of the pharyngeal repair and absence of aspiration (25, 26).

### 3.6 Early detection, public awareness, and clinical recommendations

Although TGDC occurs in up to 7% of the population, diagnosis is often delayed, especially in atypical cases at the tongue base or supraglottic region (12, 27). In the present case, the patient was aware of the tongue nodule since adolescence but did not seek evaluation until progressive dysphagia developed at age 26, representing a delay of approximately 8–16 years.

Based on this case, we suggest the following measures to reduce diagnostic delay. Primary care physicians should include routine examination of the neck and tongue base in pediatric and young adult patients; persistent midline masses warrant ultrasound evaluation and specialist referral (28, 29). Dentists and dental hygienists may also identify oral cavity masses incidentally during routine check-ups (30). Otolaryngologists and oral surgeons should maintain a high index of suspicion for synchronous lesions along the entire thyroglossal tract and perform comprehensive imaging when any tongue base or midline neck mass is identified (1, 5). Radiologists should systematically evaluate the full midline neck when incidental suprahyoid cystic lesions are detected (1, 20). We also recommend that patients and caregivers be counseled that any persistent midline neck mass—even when asymptomatic—merits evaluation, given the risks of infection, fistula, and rare malignant change (3, 31).

### 3.7 Limitations

This report has several limitations worth noting. First, the follow-up period of 18 months is insufficient to exclude recurrence of either lesion. For TGDC, recurrence after the Sistrunk procedure has been reported at rates of approximately 3%–5%, and late recurrence is well documented (14). Long-term surveillance, including periodic clinical examination and imaging, is therefore warranted. Second, no molecular or immunohistochemical evidence was obtained to support the proposed shared embryologic origin. The proposed embryologic link is speculative and cannot be confirmed from a single case. Third, this is a single case report, and the generalizability of the findings is inherently limited. A larger series would be required before any firm conclusions can be drawn.

### 3.8 Conclusion

This case represents a rare clinical presentation of a concurrent suprahyoid TGDC and lingual osseous choristoma in a 26-year-old female patient. Their coexistence suggests that both lesions may arise from the same embryologic remnant—one forming a cyst, the other differentiating into ectopic bone—at different points along the thyroglossal duct–branchial arch axis. This perspective offers a new way of thinking about the pathogenesis of lingual osseous choristoma and suggests that thyroglossal duct–related anomalies should be included in the differential diagnosis of posterior tongue base osseous lesions. Clinically, when a bony lesion is identified at the tongue base, a comprehensive radiologic evaluation of the entire thyroglossal duct tract should be performed to assess the presence of synchronous or occult TGDC. Greater awareness among clinicians is needed to reduce diagnostic delays for atypical TGDC and tongue base lesions; early surgery remains the mainstay of treatment to prevent complications and preserve function.

## 4 Patient perspective

The patient reported marked relief of the foreign-body sensation and dysphagia after surgery and expressed satisfaction with the functional recovery and overall outcome. Written informed consent for publication of this case report and accompanying images was obtained from the patient.

## Data Availability

The raw data supporting the conclusions of this article will be made available by the authors, without undue reservation.

## Supplementary material

Supplementary Figure S1 Anatomical landmarks of the posterior tongue base. Schematic illustration of the dorsal tongue emphasizing the foramen cecum and surrounding structures (e.g., vallate papillae, lingual tonsil, vallecula), which represent the most frequent anatomical region for lingual osseous choristoma in reported cases.

Supplementary Figure S2 Embryologic descent of the thyroid primordium and regression of the thyroglossal duct. (A) Fourth week: the thyroid primordium originates at the foramen cecum as a thyroid diverticulum. (B) Seventh week: caudal migration of the thyroid gland along the midline with the thyroglossal duct temporarily persisting and beginning involution. (C) Tenth week: near-complete regression of the thyroglossal duct; persistence of epithelial remnants may later give rise to thyroglossal duct cysts anywhere along the tract.

Supplementary Figure S3 Schematic of the thyroglossal duct–hyoid axis in the adult neck. Schematic diagram illustrating the course of the thyroglossal duct from the tongue base to the thyroid gland, with the suprahyoid/retro-hyoid region marked as a typical site of TGDC in adults.
