# Supplementary figures and images for: Case Report: Synchronous lingual osseous choristoma and suprahyoid thyroglossal duct cyst: insights into the embryological thyroglossal-hyoid axis
Source: Front Surg. 2026 Jul 17;13:1856644. doi: 10.3389/fsurg.2026.1856644 (PMC13423986; doi:10.3389/fsurg.2026.1856644)

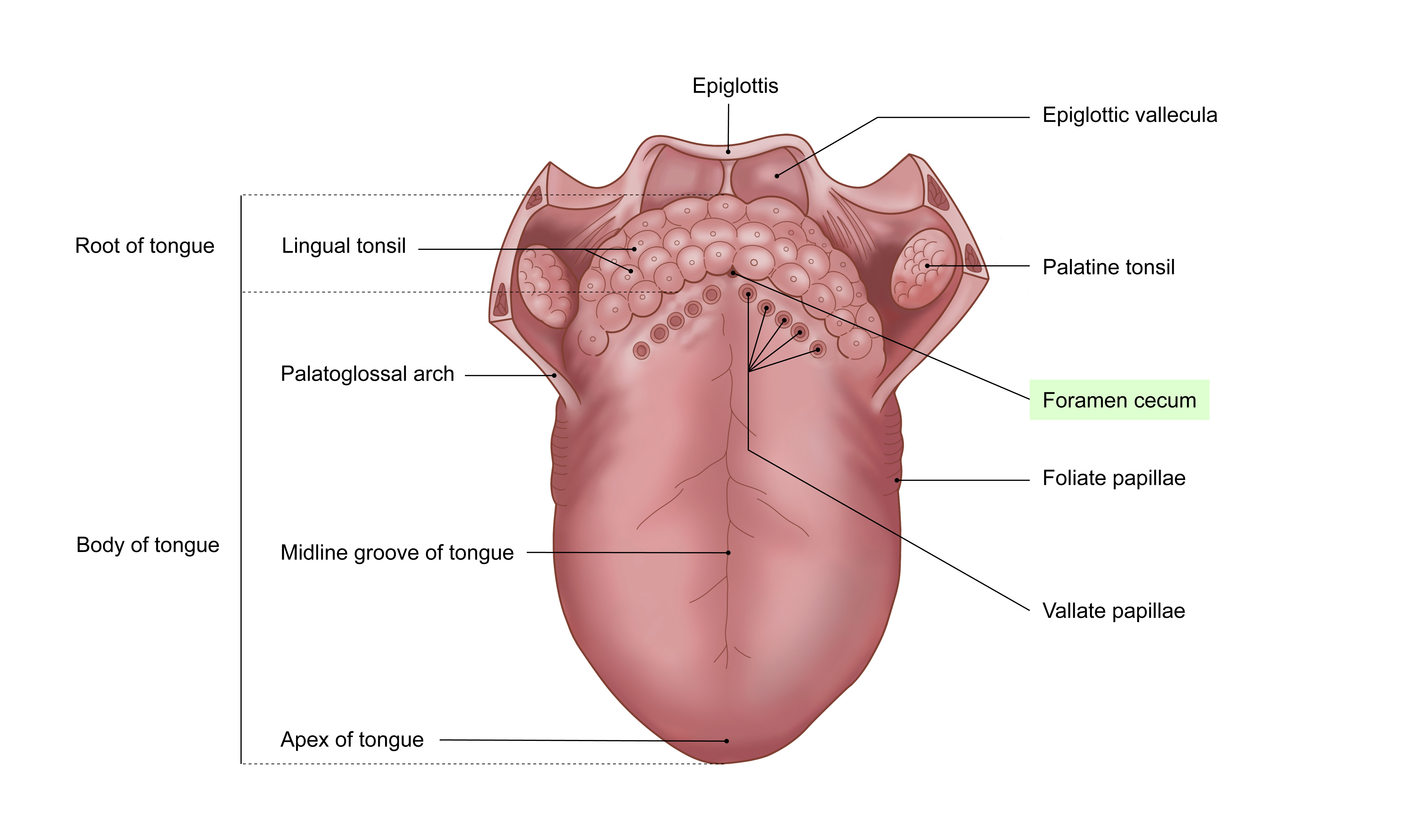

Supplement: Supplementary Figure S1 — Anatomical landmarks of the posterior tongue base. Schematic illustration of the dorsal tongue emphasizing the foramen cecum and surrounding structures (e.g., vallate papillae, lingual tonsil, vallecula), which represent the most frequent anatomical region for lingual osseous choristoma in reported cases. [file Image1.jpeg]

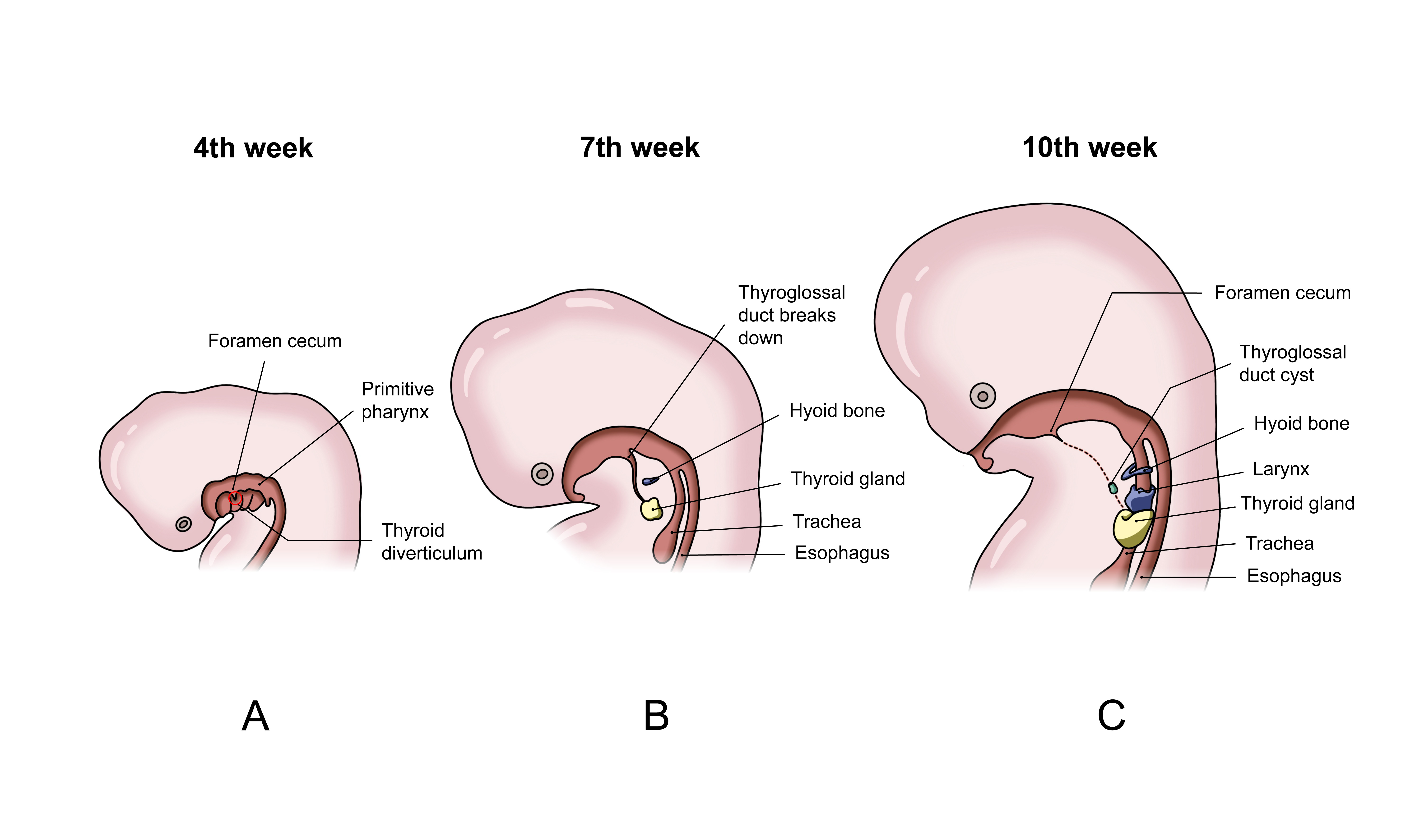

Supplement: Supplementary Figure S2 — Embryologic descent of the thyroid primordium and regression of the thyroglossal duct. (A) Fourth week: the thyroid primordium originates at the foramen cecum as a thyroid diverticulum. (B) Seventh week: caudal migration of the thyroid gland along the midline with the thyroglossal duct temporarily persisting and beginning involution. (C) Tenth week: near-complete regression of the thyroglossal duct; persistence of epithelial remnants may later give rise to thyroglossal duct cysts anywhere along the tract. [file Image2.jpeg]

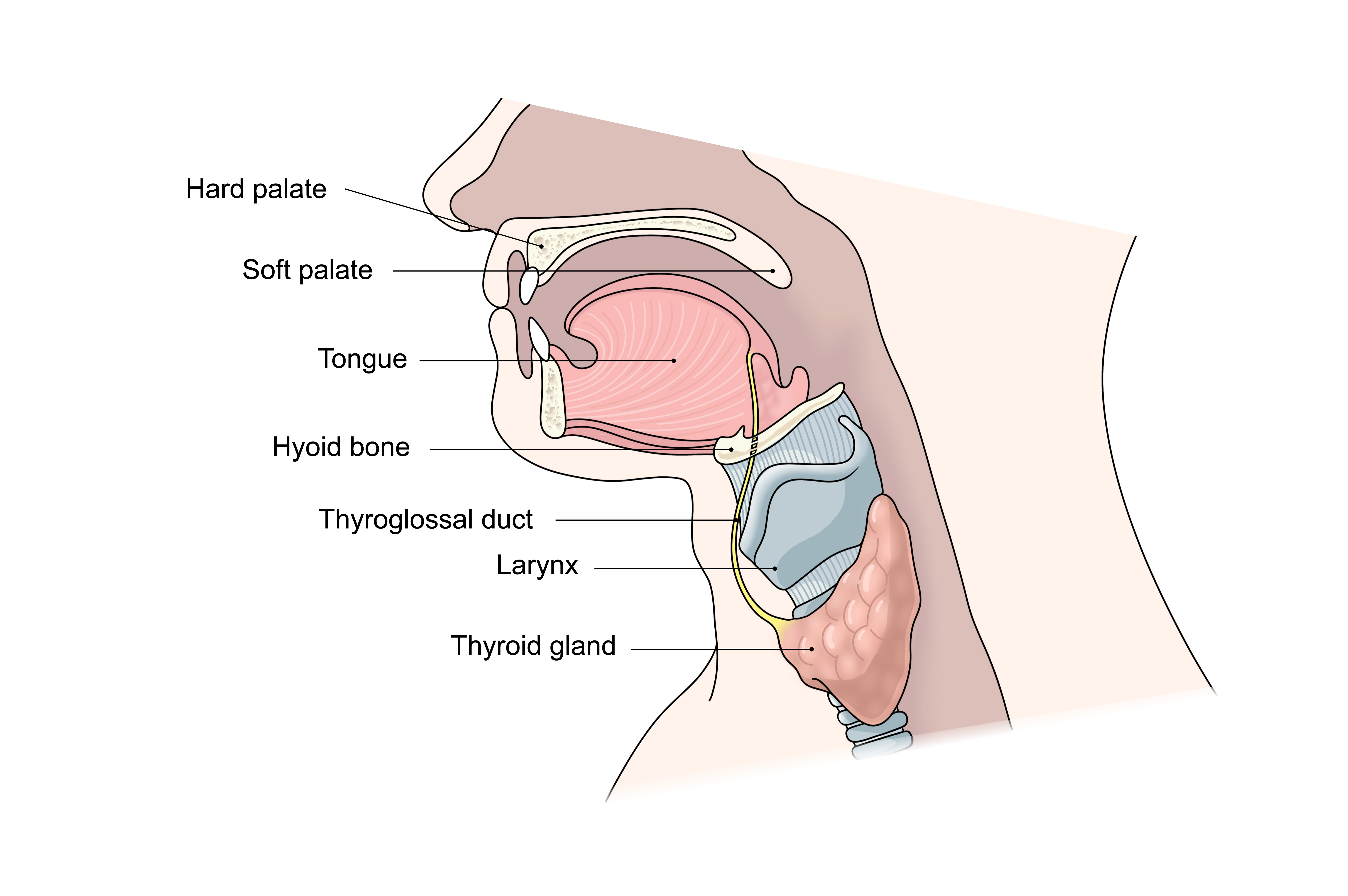

Supplement: Supplementary Figure S3 — Schematic of the thyroglossal duct–hyoid axis in the adult neck. Schematic diagram illustrating the course of the thyroglossal duct from the tongue base to the thyroid gland, with the suprahyoid/retro-hyoid region marked as a typical site of TGDC in adults. [file Image3.jpeg]
